# Lensfree Air-Quality Monitoring of Fine and Ultrafine Particulate Matter Using Vapor-Condensed Nanolenses

**DOI:** 10.1021/acsanm.3c01154

**Published:** 2023-06-14

**Authors:** Maryam Baker, Florian Gollier, Jeffrey E. Melzer, Euan McLeod

**Affiliations:** University of Arizona, Wyant College of Optical Sciences, Tucson, Arizona 85721, United States

**Keywords:** air-quality monitoring, vapor-condensed film, lensfree microscopy, nanolenses, digital holography, ultrafines

## Abstract

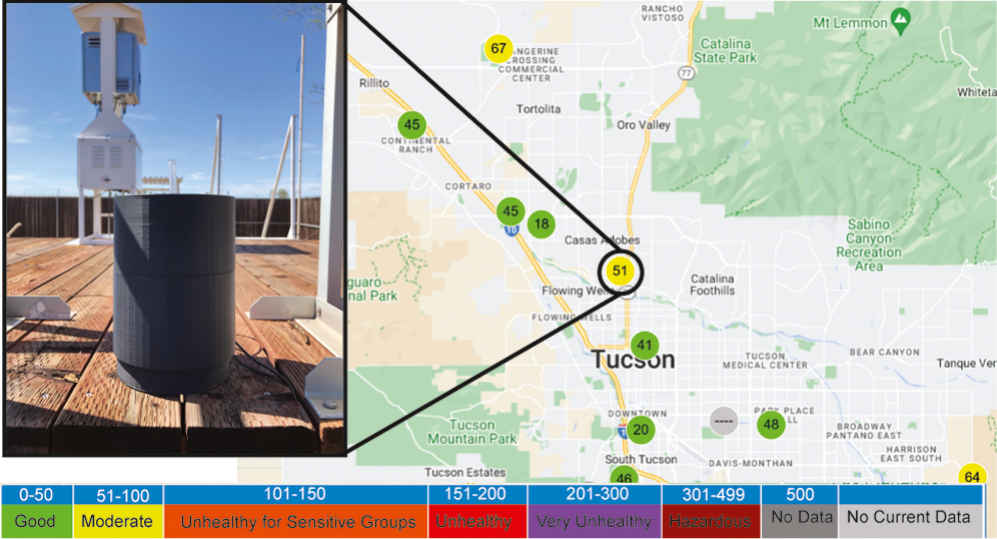

Current commercial air-quality monitoring devices lack a large dynamic range, especially at the small, ultrafine size scale. Furthermore, there is a low density of air-quality monitoring stations, reducing the precision with which local particulate matter hazards can be tracked. Here, we show a low-cost, lensfree, and portable air-quality monitoring device (LPAQD) that can detect and measure micron-sized particles down to 100 nm-sized particles, with the capability to track and measure particles in real time throughout a day and the ability to accurately measure particulate matter densities as low as 3 μg m^–3^. A vapor-condensed film is deposited onto the coverslip used to collect particles before the LPAQD is deployed at outdoor monitoring sites. The vapor-condensed film increases the scattering cross section of particles smaller than the pixel size, enabling the sub-pixel and sub-diffraction-limit-sized particles to be detected. The high dynamic range, low cost, and portability of this device can enable citizens to monitor their own air quality to hopefully impact user decisions that reduce the risk for particulate matter-related diseases.

## Introduction

The effects of air pollution on human health is of growing concern^1^ as studies show that daily exposure to particulate matter (PM) in air can have long-term adverse effects such as decreased cognitive ability,^2^ Alzheimer’s,^3^ lung cancer,^4,5^ and cardiovascular diseases.^5,6^ PM is described by the diameter of the particle, with PM_10_ or “coarse particles” used to describe particles with diameters of 10 μm or less, PM_2.5_ or “fine particles” to describe particles with diameters of 2.5 μm or less, and PM_0.1_ or “ultrafine particles” to describe particles with diameters of 100 nm or less.^7^ Previously, many studies focused on the health effects of PM_10_ and PM_2.5_, attributing 1 out of every 9 deaths in 2012^8^ and 8.9 million deaths in 2015 to fine particulate matter air pollution.^9^ However, compared to coarse and fine particles, ultrafine particles (UFPs) have distinct dynamics inside and outside the body,^10^ contributing to growing evidence that more attention should be paid to detecting and quantifying UFPs due to their potentially higher toxicity. Inside the body, UFPs can penetrate the alveolar–capillary barrier, enabling them to travel through the body by way of the circulatory system and can linger for months after exposure.^11^ Due to their higher specific surface area (total exposed surface area per unit of mass), UFPs can also adsorb greater quantities of hazardous metals and organic compounds that cause oxidative stress or the inability to detoxify inhaled reactive products.^12^ Outside the body, UFPs have dynamics that can cause them to bypass filtration and coagulate with other particles. In urban areas, the main source of ambient UFPs is from motor vehicles.^13,14^ Although a small subsection of motor vehicles, many diesel vehicles have particulate filters to reduce the emitted particulate matter. However, many UFPs are formed after filtration by condensation of gas exiting the tailpipe,^15,16^ making these filters ineffective for UFP filtration. UFPs can also decay quickly and do not travel far beyond the source of emission,^16^ which makes detecting and quantifying UFPs at sparsely distributed ground stations difficult.

As of August 2020, there are more than 30,000 known air-quality monitoring stations throughout the world with at least 12,000 publishing data on PM_10_ and PM_2.5_ measurements on the World Air Quality Index project.^17^ While a growing effort, the percentage of inhabited land with air-quality monitoring stations is still small, and many of these stations are far from smaller indoor and outdoor environments frequented by people.^6^ Historically, particulate matter has been measured using particle mass concentration and particle number concentration.^18^ There are two main methods of measuring particulate matter at air-quality monitoring ground stations: β attenuation monitoring (BAM) and tapered element oscillation microbalance (TEOM). For BAM, the β gauge provides mass concentration by measuring the attenuation of β radiation (high energy electrons) after passing through collected particulate matter,^19^ while the TEOM measures mass concentration by measuring changes in the resonant frequency of a hollow glass tube as collected particles change the weight of the filter in the device.^20^ These devices are stationary, bulky, and can cost upward of $5000.^21^

There are also devices that are able to provide real-time measurements of PM_0.1_ such as a scanning mobility particle sizer (SMPS), an electrical low-pressure impactor (ELPI), and commercial devices such as the NanoMOUDI-II Impactor, and the QCM-MOUDI Impactor. SMPSs are stationary devices that flow particles into the device and size them using a differential mobility analyzer that sizes the particles based on mobility through an electric field. Particles are then selected based on their mobility and are flowed through a condensation particle counter to determine particle concentration for each size sequentially.^22,23^ ELPIs use cascaded impactors to separate particles by size. The particles are also charged when they enter the device, and the impactors are connected to electrometers. When particles land on the impactors, the charge is measured and used to determine the concentration of the particles at each impactor.^24,25^ Other impactor-based devices include the NanoMOUDI-II and QCM-MOUDI impactors. The NanoMOUDI-II uses a rotating impaction plate to uniformly deposit particles of a range of sizes.^26^ The QCM-MOUDI uses a quartz crystal microbalance (QCM) to determine the number of particles on each of the six impactors used to collect particles of different sizes.^27^ While accurate, these devices are bulky and remain expensive.^24,26,27^

The shortcomings of current air-quality monitoring devices, such as high cost, low portability, and low density (number of air-quality monitoring devices per number of people), have fueled many alternative portable low-cost air-quality monitoring devices to enable people to monitor their personal exposure levels^28,29^ based on their unique habits and locations. Many of these low-cost devices integrate smartphone technology and machine learning to image collections of PM.^30−32^ However, these smartphone-based devices are not able to perform long-term monitoring in the same area, as people travel with their phones throughout the day.

An alternative with comparable resolution is a lensfree holographic device. Lensfree holographic devices utilize computational imaging to post-process images of diffraction patterns collected on the sensor to form an image.^33,34^ With no lens and a close sample-to-sensor distance, lensfree holographic devices are in a unit magnification configuration enabling imaging over the whole sensor. This results in images with resolution comparable to lens-based systems over the whole field of view of the sensor. Previously, lensfree holographic devices have been used for air-quality monitoring^35^ and volatile aerosol characterization of e-cigarette smoke.^36^ However, currently, both these smartphone-based and lensfree holographic devices are limited in resolution to particles larger than 1 μm,^30−32,35^ or in some cases, particles larger than 500 nm.^36^ In some of our prior work, we have optimized lensfree holographic microscopy for nanoparticle detection, but have not applied this to aerosolized particulate matter sensing.^37−41^

Other optical-based systems measure the change in transmission as particles adhere^42^ or pass through^43^ a fiber and can detect particles smaller than 1 μm. Another laser-based system uses heterodyne detection to measure the change in interference when nanoparticles pass through one of the beam paths.^44^ However, these devices require lasers, which are often neither low-cost nor easily portable. Some other nonimaging-based low-cost portable devices are able to detect particles smaller than 500 nm by measuring charged particle mobility^45,46^ or change in transmission through a silicon-on-insolator waveguide when nanoparticles adhere to the surface.^47^ However, these devices are not as easily replicable by citizen scientists as lensfree holographic systems, which often only require a commercial sensor, light source such as an LED, and a 3D-printed housing for which files can be made easily accessible.

Here, we report a holographic lensfree portable air-quality monitoring device (LPAQD) that can detect and quantify PM_10_ down to PM_0.1_ in the same device (Figure 1). The LPAQD is a low-cost (∼$325), portable, and easily replicable lensfree holographic system with real-time tracking capability that uses a vapor-condensed nanofilm to improve the signal-to-noise ratio (SNR) of particles smaller than the pixel size of the detector. The signal is considered to be the average pixel value corresponding to a particle in the lensfree hologram, and the noise is the average pixel value of the portion of the lensfree hologram that does not have any particles (the background). Previously, PEG nanofilms have been deposited over particles to increase the scattering cross section and SNR of particles smaller than the pixel size.^40,41,48^ In our experiments, we instead vapor-condense the PEG *before* the particles are deposited, achieving the same nanolensing effect as before but now with the ability to track particle accumulation in real time by acquiring a sequence of images over time. In the previous approaches, all of the particles on the slide had to be imaged at the same time at the end of the experiment, after collection was finished. By acquiring real-time particle accumulation data, air quality hazards can be identified when they happen, rather than many hours or days after the event.

## Materials and Methods

**Figure 1:**
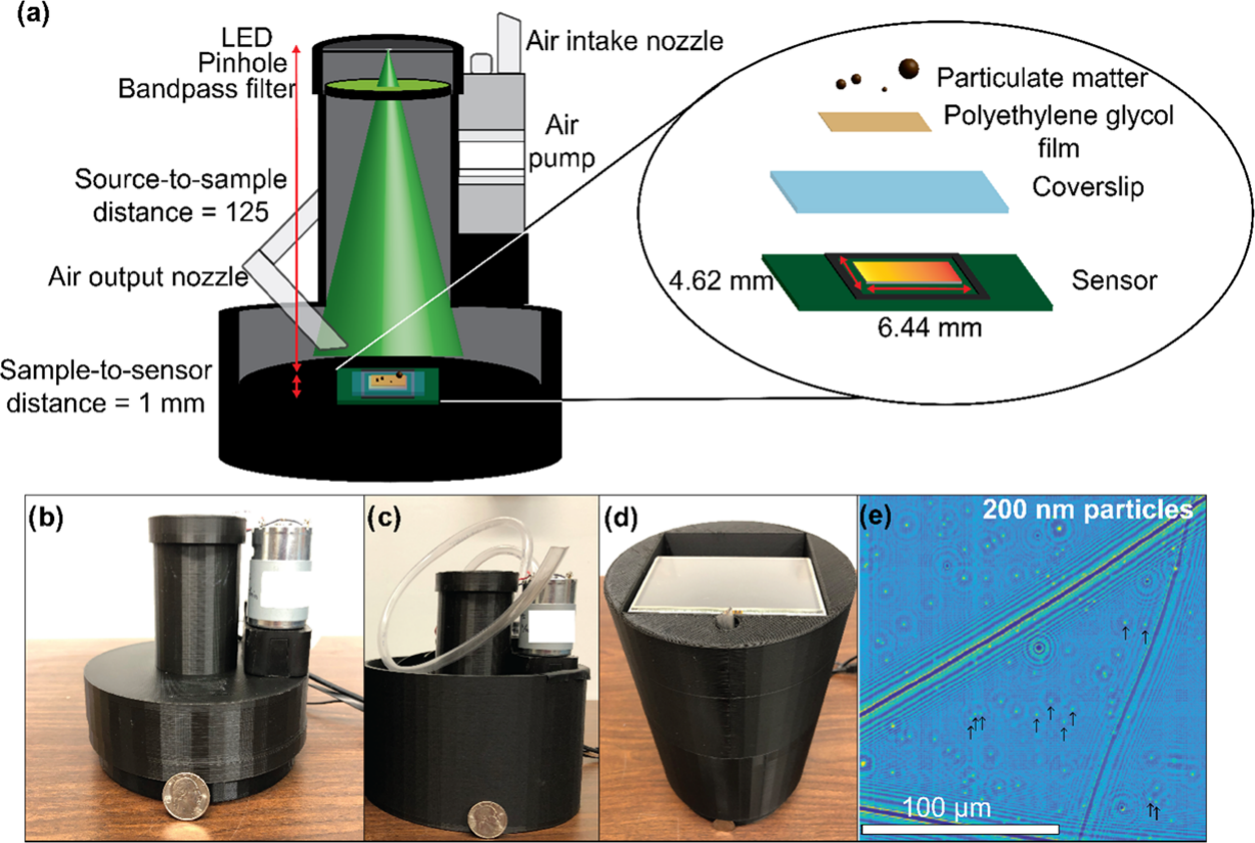
Portable lensfree air-quality monitoring device. (a) Diagram of the system. (b–d) Photographs of physical hardware. (b) Main device with air pump. (c) Main device and pump in the bottom portion of housing. (d) Main device and pump completely enclosed in housing. The LCD display on the device shows particulate matter as deposited onto the PEG film in real time. (e) Reconstructed hologram of a diffraction pattern generated by 200 nm spherical nanoparticles deposited onto a PEG film using the device. Arrows show some of the 200 nm particles.

### LPAQD

The LPAQD is composed of an LED, a pinhole, a bandpass filter, a camera sensor, and 3D-printed housing. The LED (Golden DRAGON λ = 528 ± 15 nm) is spatially filtered by a pinhole (250 μm diameter) and temporally filtered by a 530.5–533.5 nm bandpass filter (Thorlabs FL532-3) and soft-coated with a minimum transmission of 60% at the center wavelength, to achieve a partially coherent plane wave at the sensor plane. The camera sensor (Arducam MT9J001) is monochrome with a pixel size of 1.67 μm. The coverslip with a vapor-condensed film on the top plane is then placed directly on the sensor, with the bottom plane facing the sensor. The sensor still has the manufacturer’s protective cover glass attached, so the distance between the sample and the sensor is ∼1 mm. The real-time tracking is enabled by a Raspberry Pi 3 and an LCD screen (ELECROW 5” Raspberry Pi Screen).

### Polyethylene Glycol (PEG) Deposition

**Figure 2:**
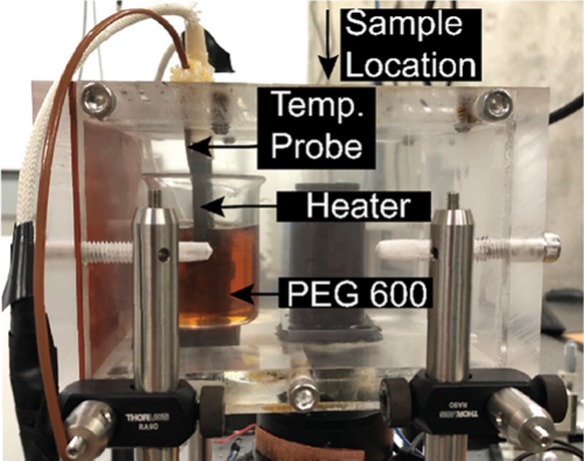
Device used to condense PEG onto coverslip before being placed in the LPAQD.

PEG 600 is vapor-condensed onto a plasma-treated coverslip (Globe Scientific) using the system shown in Figure 2. The coverslip is placed at the opening on top of the system, and a room-temperature aluminum plate is placed on top of the coverslip to ensure uniform condensation. The PEG is heated to 140 °C in a beaker using a U-shaped heater. The temperature of the heater is maintained by a proportional-integral-derivative (PID) temperature controller driven by a LabVIEW program, which also monitors the temperature of the PEG from a temperature probe to ensure proper performance of the PID loop. PEG is deposited for 5 min, resulting in a thickness of ∼110 nm. After the PEG was deposited, the film thickness was measured using an ellipsometer (M-2000 J. A. Woollam).

**Figure 3:**
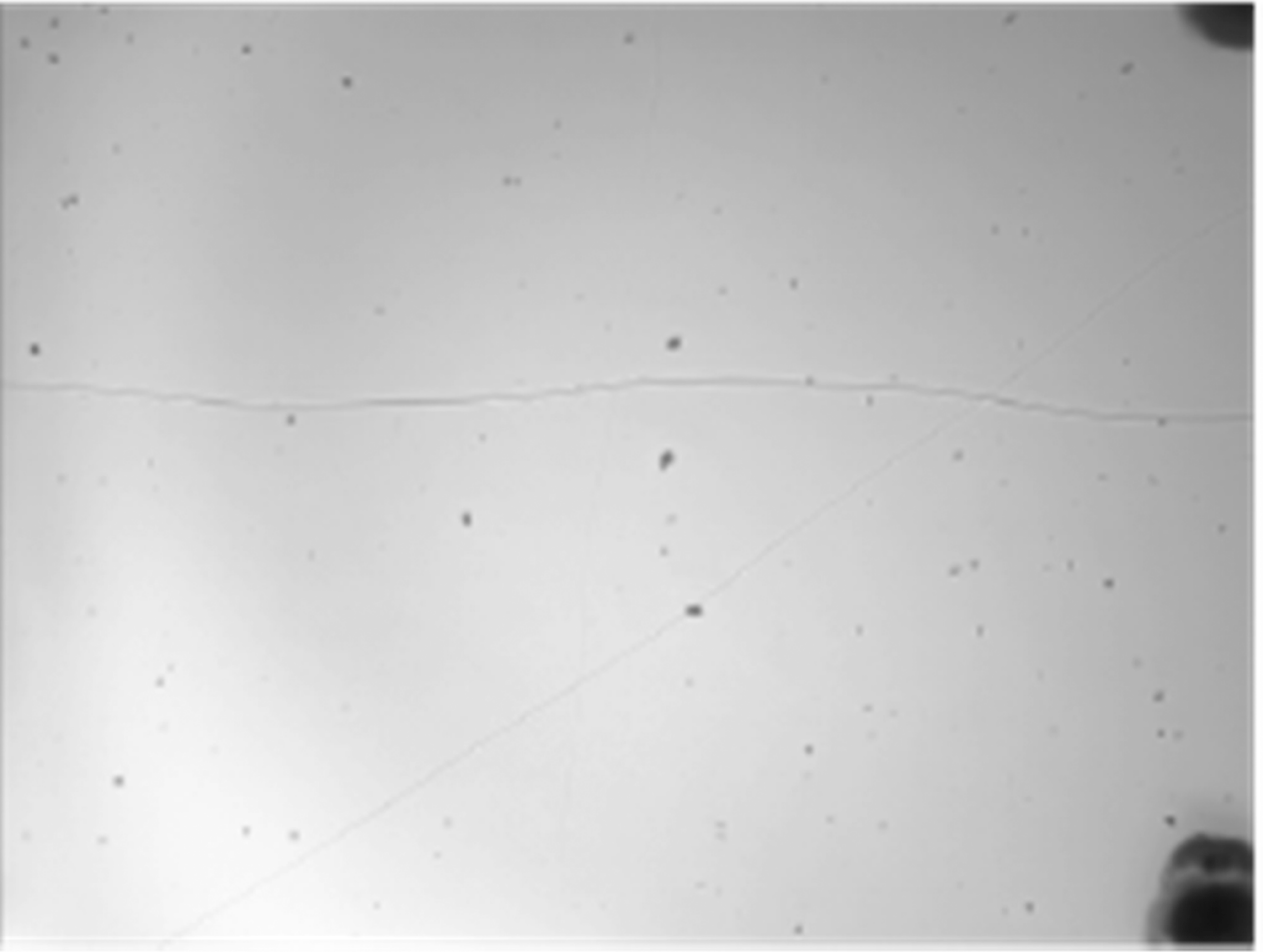
Lensfree image taken with the LPAQD during an outdoor air-quality monitoring session, showing collected particulate matter. The lines are scratches on the backside of the coverslip, where PEG is not deposited, and used as a reference between images. The large dark clumps in the center of the image are examples of debris collected by the device.

### Aerosolization of Test Particles

The device was initially tested by aerosolizing fluorescing particles of known size into the device using an aerosol generation system (Brechtel Manufacturing Inc. 9200). Once the fluorescing particles were aerosolized onto a coverslip with PEG, lensfree images were taken with the LPAQD, and reconstructed particles were identified. Solutions with fluorescent spherical particles of known diameters (1 μm, 500 nm, 300 nm, 200 nm, and 100 nm) were diluted and placed into the aerosolizer, which blew the particles into the LPAQD, where they were captured on the PEG film. The solutions were prepared by depositing microliter amounts of monodisperse (coefficient of variation <10%) fluorescing particles into milliliter amounts of deionized water. Once the fluorescing particles were aerosolized onto a coverslip with PEG, lensfree images were taken with the LPAQD, and reconstructed particles were identified. Particles found in the lensfree reconstruction were validated with images of the same coverslip in a benchtop fluorescence microscope for particles of sizes between 1 μm and 200 nm, and a scanning electron microscope for 100 nm particles.

### Air Flow and Data Acquisition

Air is pulled into the LPAQD using an aquarium air pump (AIRPO) with a flow rate of 4.5 L/min. The device does not use an impactor, and the outtake nozzle of the air pump is placed in a hole at the top of the device to direct the air toward the coverslip while staying out of the field of view of the camera. An impactor is not used as it would be in the imaging path while taking real-time measurements when deployed at a testing site. Also, the goal for this device was to sample a portion of the intake air in order to extend the sampling life of the device by not saturating the deposited film within hours of device deployment.

To monitor the particulate matter collected over time, an LCD screen connected to the camera in LPAQD, Figure 1d, displays one of 20 lensfree images taken every 10 min. An example is shown in Figure 3. The lines are scratches on the backside of the coverslip, where PEG is not deposited and used as a reference between images. The large dark clumps in the center of the image are examples of debris collected by the device. The camera is controlled using a Python code run by a Raspberry Pi 3 and is programmed to take 20 images, which are later averaged every 10 min.

### Nanolensing

**Figure 4:**
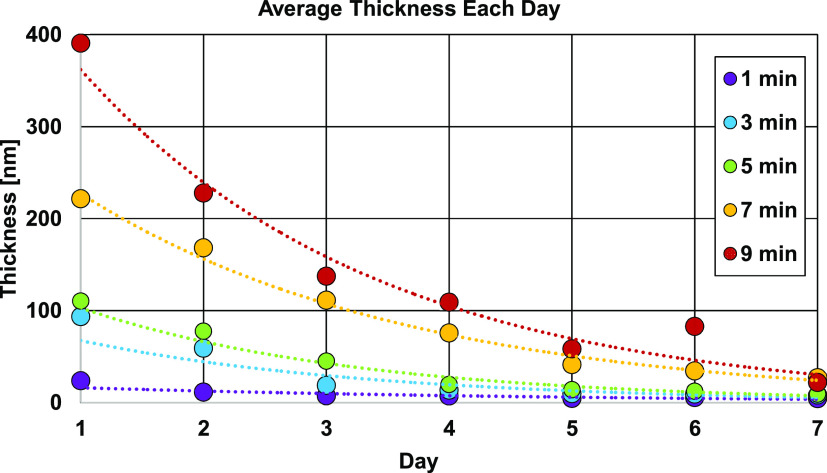
Evaporation of different thicknesses of PEG 600 films. Dotted lines show exponential fits to data. The legend shows the deposition times.

This device uses nanolensing to improve the SNR of sub-pixel-sized particles in the lensfree hologram. The nanolenses are formed when the particles are deposited onto a vapor-condensed PEG film and the film deforms, creating a meniscus along the surface of the particle.^48^ Many liquids could be used to form nanolenses around particles. We used PEG because it is nontoxic, nonvolatile, and its liquid state yields a smooth surface. In particular, we selected PEG 600 instead of the previously used PEG 400^48^ for its higher vapor pressure at room temperature so that the film would be “shelf-stable” over days, as shown in Figure 4. PEG 600 is also ideal for vapor condensation as it is easy to achieve a thin film across a coverslip, demonstrated by the appearance of fringes due to thin-film interference, when heated. The film is hydrophilic and can be removed with water; however, the film is protected inside the LPAQD during testing.

### Image Reconstruction

Images were reconstructed using the angular spectrum method (ASM), which is common in lensfree digital holography. The ASM decomposes a field into a superposition of plane waves that are traveling at different angles, as described by eq 1.
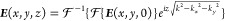
1The spatial frequency components along *x* and *y* are described by *k*_*x*_ and *k*_*y*_, *k* is the wavenumber, *z* is the propagation distance, and  and  describe the Fourier and inverse Fourier transformations. The ASM is used for field propagation, propagating a field at *z* = 0 to some distance *z*, using the transfer function of free space, which is multiplied by the Fourier transform of the initial field.

In lensfree holography, scalar fields are assumed, and so ***E*** becomes *E*. To perform the image reconstruction, light detected at the sensor plane, *E*(*x*, *y*, 0), is back-propagated to the object plane, *E*(*x*, *y*, *z*), to reconstruct the object field, where *z* is the negative of the object-to-sensor distance. The reconstruction is done over the whole field of view of the sensor, making high-resolution, large field-of-view reconstructions achievable in a single step.

### Particle Sizing and Counting

The particles are automatically detected, sized, and counted using a previously published image processing approach, written in MATLAB.^40^ The code sizes and counts the particles in the image reconstructed using ASM based on the phase of the identified particles. The image processing MATLAB code uses the phase values in lensfree reconstructions to detect and size particles. The image processing uses the averaged lensfree images taken during testing. First, the user defines a background value by selecting a portion of the lensfree image where there are no particles. Next, the code uses eq 1 to back-propagate the lensfree image using an approximate back-propagation distance specified by the user. The precise back-propagation distance is found in the code by back-propagating the image until the phase value of a particular particle is maximized. The phase values of the detected particles are stored, and then the particles and their twin images are removed from the raw lensfree image to reduce optical fringe artifacts that could obscure smaller particles. The code then back-propagates the new lensfree image, where only smaller particles remain in the image, and the process is repeated for a smaller threshold to count and size the next-smaller range of particles. The code repeats this process for five decreasing threshold values, which correspond to phase values of the reconstruction, and then uses a calibration file that maps the recorded phase values to particle sizes. The calibration file is generated from measured phase values (see the Results and Discussion section) fit to eq 2,(40) where *D* is the particle diameter, ϕ_pk_ is the measured peak phase, and *A*, *B*, and *C* are fitted parameters. The peak phase of particles of known size was measured over days and used to determine particle size in the image reconstructions.
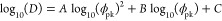
2Once the particles are sized, a histogram is generated. The LPAQD is an integrative sensor, so each hour more particles are collected onto the film. The PM level is determined each hour by measuring the change in the number of particles with respect to the previous hour.

### Outdoor Testing

The LPAQD was taken to the Pima County testing site at the Children’s Park to perform outdoor air-quality monitoring. Images taken at each hour were processed to obtain the number of particles counted over the previous hour and determine the corresponding size distributions. The testing site was along a highly frequented bike path and ∼300 m from a major road.

## Results and Discussion

To determine the optimum deposition time and thickness to form nanolenses around 1 μm and 100 nm particles simultaneously, the thickness of vapor-condensed PEG films was measured for various deposition times using an ellipsometer. Since PEG evaporates, the thickness was measured over a week to determine the “shelf-stability” of the PEG film (Figure 4).

It was determined that a 5 min deposition was optimal for forming nanolenses around particles based on nanolens formation theory from previous work done on vapor-condensed nanolenses.^48^ In previous work, the film was vapor-condensed onto particles placed directly onto a coverslip as opposed to particles being deposited onto a coverslip with a vapor-condensed film. The lens shape formed by the particle covered in the PEG film is governed by surface tension and was modeled using the Young-Laplace equation, accounting for the van der Waals disjoining pressure.^48^ The lens formation model can predict the optimal film thickness for generating the largest phase value in the lensfree reconstruction, for a range of spherical particle diameters. The film thickness was optimized to image the 100 nm particles since peak phase detection is more demanding for smaller particles, as bigger particles can be more easily detected even without any film, and it does not take much for small particles to become buried under the film. In previous work, it was found that the optimal film thickness was approximately equal to the bead radius.^40^ Extending this to 100 nm particles, a 50 nm film thickness is best for nanolensing. We wanted to make sure we could deposit the PEG film a day or two before testing so that in future iterations of the device, users would not need to deposit a PEG film each day for data acquisition but could instead have a stock of coverslips with predeposited film. So, we determined that the PEG deposited for 5 min was ideal, since it maintained a thickness of around 50 nm for a day or two.

The PEG thickness decayed due to gradual evaporation each day, except for the case of the PEG film deposited for 9 min as seen by a measured thickness increase on day six. This blip is most likely an artifact of film rupture and reorganization into droplets.^49−51^

**Figure 5:**
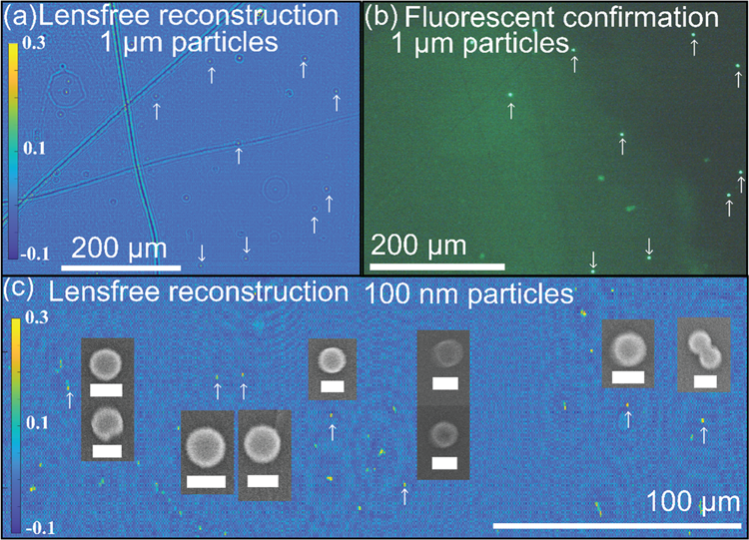
Evidence of nanolensing for 1 μm and 100 nm spherical nanoparticles. (a) A small region of interest of a reconstructed hologram from the diffraction pattern of 1 μm particles aerosolized into the LPAQD and deposited on the PEG film. (b) Confirmation of location of 1 μm particles with an image from a fluorescence microscope of the same sample. (c) Reconstructed hologram from the diffraction pattern from 100 nm spherical nanoparticles aerosolized in the device and deposited on PEG film. Insets show images of the same particles in an SEM to confirm the locations of the 100 nm particles. The scale bars in the SEM images are 100 nm.

Particles of known sizes were aerosolized in the device and onto the PEG-coated coverslip to determine the smallest particle size that could be detected. Fluorescing 1 μm, 500 nm, 300 nm, and 200 nm particles were used so that particle locations in the holographic reconstructions could be confirmed against images from a fluorescence microscope. A scanning electron microscope (SEM) was used to determine the particle locations of identified 100 nm particles in the holographic reconstructions. Figure 5 shows the reconstruction and validation images of the largest and smallest particles we study here, corresponding to 1 μm and 100 nm particles.

**Figure 6:**
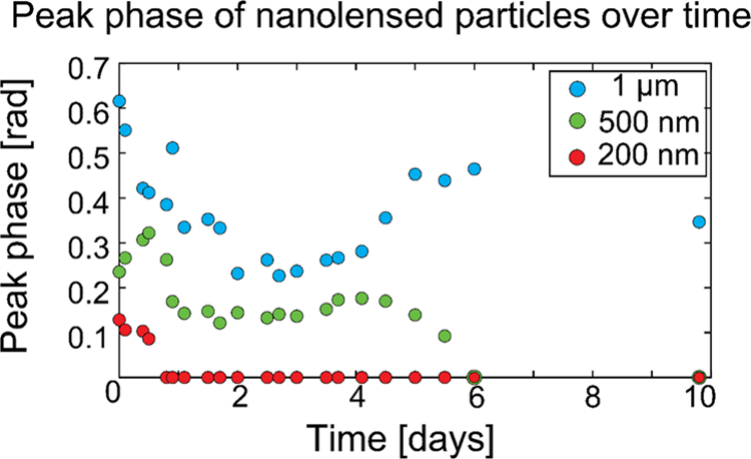
Peak phase of particles in the holographic reconstructions as the PEG evaporated.

**Figure 7:**
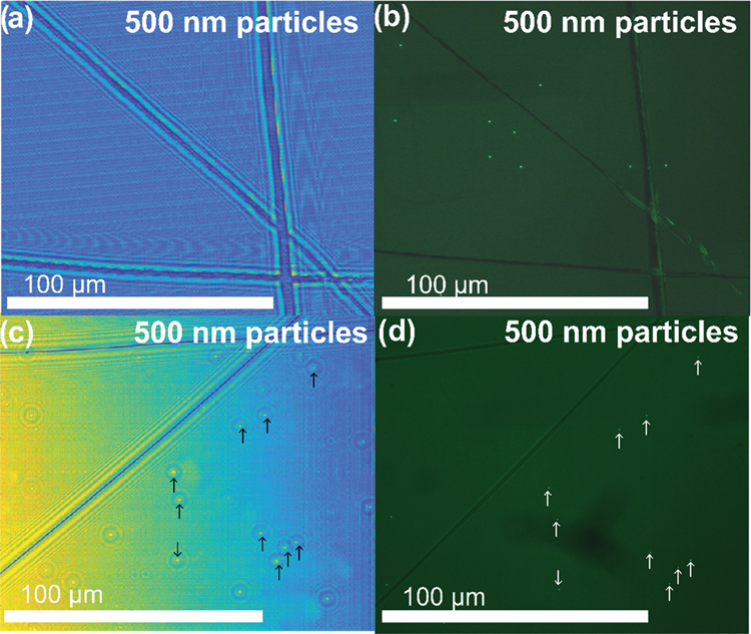
Demonstration of the nanolensing effect of PEG deposited before particles. (a) Coverslip with no PEG and 500 nm aerosolized particles. The lines in the image are scratches used as registration markers. (b) Image of the same sample in panel (a) using a fluorescence microscope, demonstrating that there are 500 nm particles present. (c) Coverslip with PEG and 500 nm aerosolized particles clearly observable. (d) Image of the same sample in panel (c) using a fluorescence microscope, validating the locations of the 500 nm particles located in the holographic reconstruction.

For particles of known sizes, lensfree images were taken over 10 days to measure the peak phase of each particle as the PEG evaporated (Figure 6) as the film thickness affects the curvature of the meniscus formed along the surface of the particle and consequently the particle’s SNR in the lensfree image. Example lensfree images are shown in Figure 7. The phase measured at time 0 was used in the MATLAB script to size and count particles of unknown size. After 1 day, the 200 nm particles were no longer visible in the reconstructions, and after 6 days, the 500 nm particles were no longer visible in the reconstructions. The SNR of smaller particles is more sensitive to changes in film thickness, such as evaporation, as a few nanometer difference in film thickness constitutes a relatively large fraction of particle diameter compared to larger particles. Figure 6 implies that the PEG film is usable for particles >200 nm for longer than a day. However, the sensitivity of particles <200 nm to the thickness of the PEG film restricts the use of the PEG film beyond a day. For the first 3 days, the peak phase of the 1 μm and 500 nm particles decreased as expected. However, on day 4, the peak phase increased. This could be related to film instability, rupture, and reorganization, which was observed in Figure 4 for the PEG deposited for 9 min. The 1 μm and 500 nm particles may serve as nucleating seeds for the film reorganization after rupture, creating a larger meniscus and enhancing the nanolensing effect.

The PEG film has a response time on the order of a few seconds, so once a particle lands on the film, a meniscus is formed almost immediately. The particles then remain and are only removed if extracted, such as by wiping or rinsing off.

Figure 6 shows the nanolensing effect due to the PEG on 1 μm, 500 nm, and 200 nm particles. Without PEG, the particles are undetectable in the lensfree image, even though their presence is confirmed by the fluorescence microscope image.

**Figure 8:**
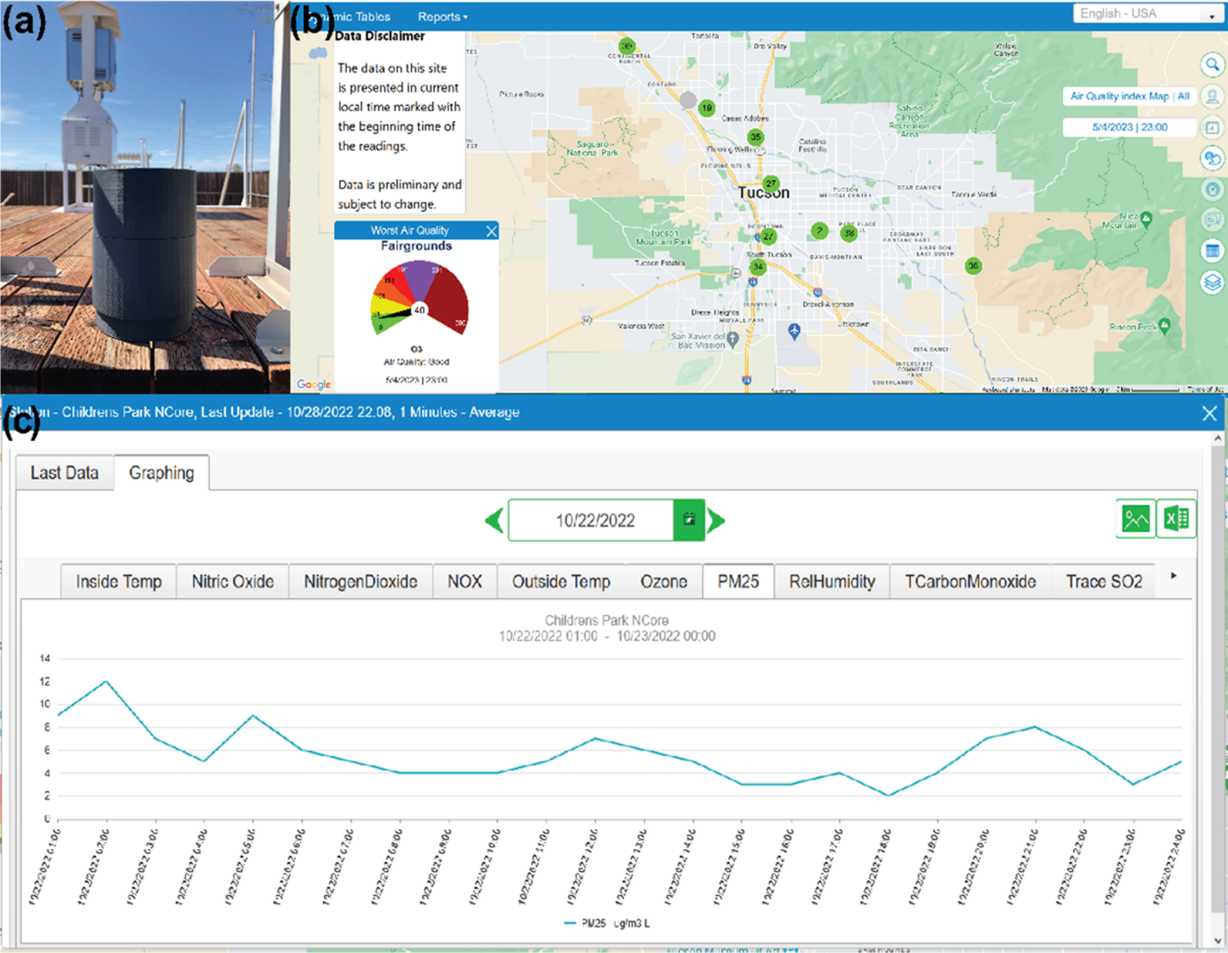
Testing the LPAQD at the Pima County Children’s Park. (a) The LPAQD at the testing site. The county BAM device is in the background. (b) Map of the city with locations of sites of air quality-monitoring stations shown as circles with color and number indicating air quality using the air quality index. (c) Published PM_2.5_ data from the county on the day the LPAQD was taken to the testing site. The blue trace of PM_2.5_ reports measurements on October 22, 2022, from 1:00 to 24:00.

The Pima County air-quality monitoring sites report the PM concentration in μg/m^3^ each hour. Figure 8 shows the correlation between the number of new particles detected each hour in the LPAQD and the PM_2.5_ hourly concentrations published by the county. The error bars in the *x*-direction show the error due to rounding since the county reports the hourly PM_2.5_ concentration as whole numbers. The error bars in the *y*-direction show the error in particle counts from the particle detection and counting code. The error is determined by minimizing and maximizing the value of the noise threshold in the code, the value above which a pixel in the holographic reconstruction is considered to be from a detected particle.

**Figure 9:**
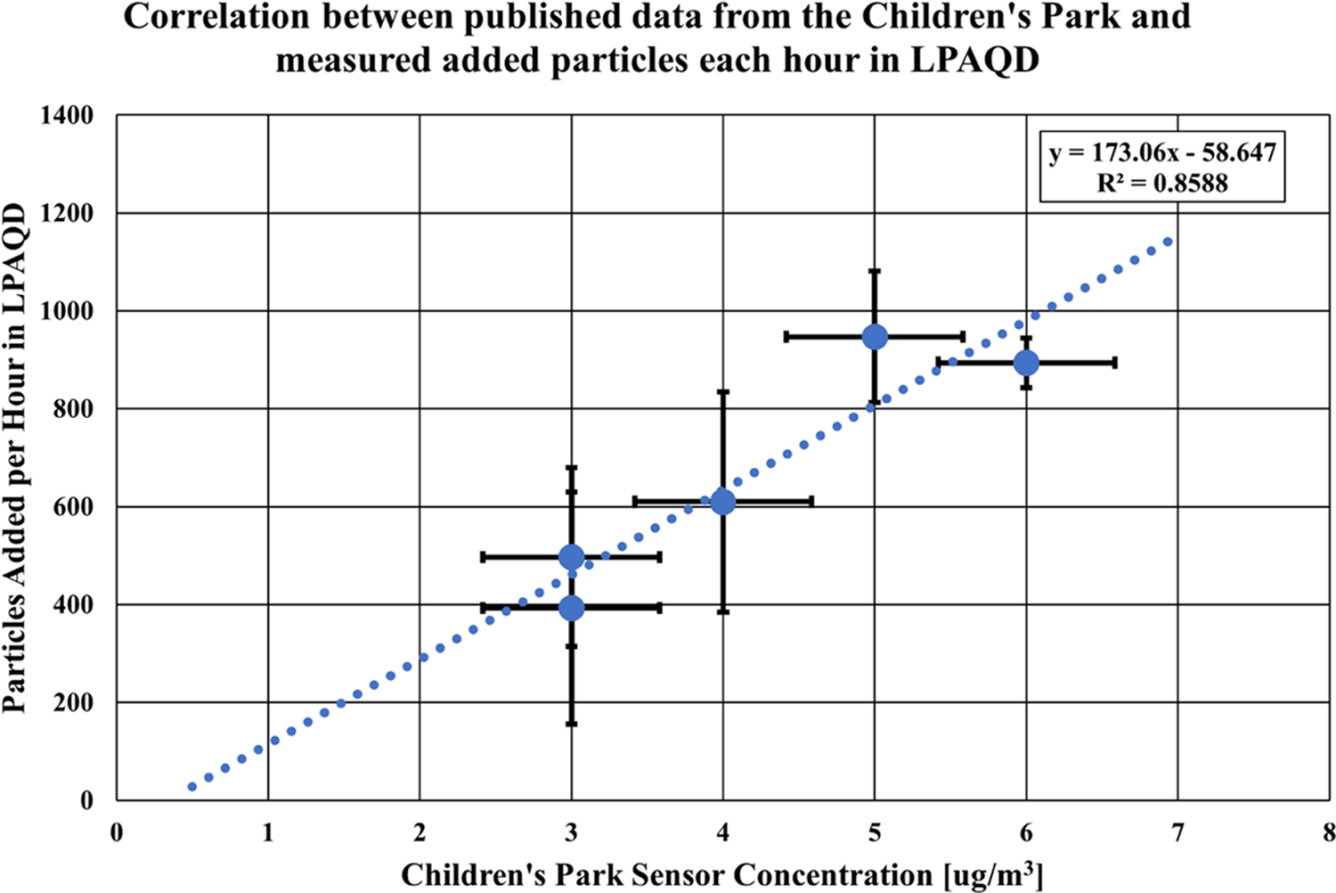
Correlation of new particles counted each hour in the LPAQD with the PM_2.5_ hourly concentrations reported by the county.

The correlation demonstrates that the LPAQD can detect a change in the number of particles each hour that correlates with the measured change in PM_2.5_ concentration from a commercial device. Since the devices measure different quantities (number of particles versus total mass of particulate matter below a cutoff threshold), it is only expected to see a correlation and not an equal measurement. However, the number of particles and particle sizes determined from the data collected by the LPAQD can be converted into a PM concentration by calculating the mass of the particles we capture on the sensor (eq 3) and then applying a collection efficiency to convert the value to the total number of particles in the air (eq 4).
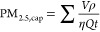
3
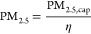
4where *V* is the particle volume, ρ is the particle density, which is assumed to be the density of polystyrene, *Q* is the volumetric air flow rate, *t* is the collection time, and η is the collection efficiency of the LPAQD, which is the ratio of particles that land on the PEG to the number of particles drawn into the device. For the LPAQD, we determined that η is 1.91 × 10^–4^ by calculating the PM_2.5,cap_ value for each point in Figure 9 using eq 3. The determined collection efficiency was then the value of η that gave the best linear fit through the trusted Children’s Park PM_2.5_ vs our captured PM_2.5,cap_ level at the same point in time. This is related to the slope in Figure 9 but takes into account the size of each captured particle. The root-mean-square error of the residuals is 0.74 μg/m^3^.

Due to the relatively good air quality in Tucson, AZ, the PM_2.5_ levels in Figure 9 are low, which serves as an excellent validation of our high sensitivity and low limit of detection. On the other hand, we were unable to field-test our device at high particulate matter levels. We can, however, estimate that with a 10-megapixel sensor where each particle occupies approximately a 10-pixel × 10-pixel region on the sensor, there is space for up to 10^5^ particles. This means that in 1 h, we could measure concentrations about 100× greater than the 6 μg/m^3^ that we sense in Figure 9, or 600 μg/m^3^, which is well beyond the highest “Hazardous” rating of PM_2.5_ levels. Thus, our sensor spans the relevant range for guidance on human exposure. We can also estimate the saturation time of the sensor, which depends on the particulate concentration levels. At a relatively low 10 μg/m^3^ PM_2.5_ concentration, saturation is expected to take ∼100 h or 4 days. At a moderately unhealthy concentration of 100 μg/m^3^, saturation is expected within ∼10 h.

**Figure 10:**
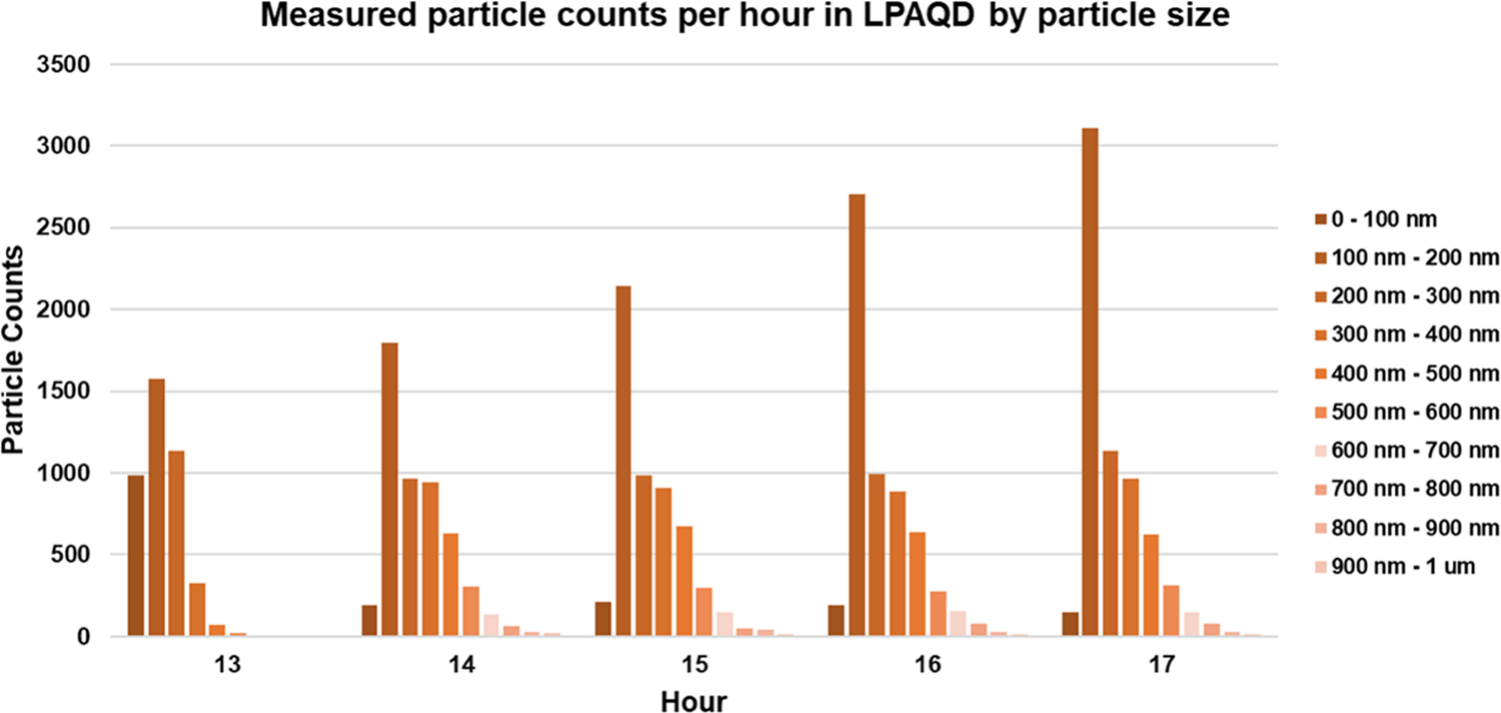
Measured PM concentration in the LPAQD by particle size for each hour.

The LPAQD detected particle sizes between 100 nm and 1 μm. Figure 10 shows the measured change in particulate concentration by particle size every hour. This information is not yet available from commercial devices and is critical for understanding PM_1_ and PM_0.1_ levels and for assessing air that could be harmful. Quantifying the PM concentration by particle size per hour also enables an understanding of sources of PM based on particle size and timing of polluting events.

To evaluate performance, we also consulted the U.S. EPA Performance Testing Protocols, Metrics, and Target Values for Fine Particulate Matter Air Sensors.^52^ The performance metric for linearity is the coefficient of determination (*R*^2^) with a target value of ≥0.70. We have demonstrated performance in accordance with these metrics with an *R*^2^ value of 0.86, as shown in Figure 9. The performance metric for precision can be given by the coefficient of variation, which has a target value of ≤30%. When the PM concentration is calculated using eq 3 and scaled using η (assuming the same η for all particle sizes), the coefficient of variation is 46%. To improve the precision in future iterations, the airflow could be improved with a more stable pump and precisely regulated flow geometry.

## Conclusions

We have demonstrated a lensfree holographic imaging device that can detect, size, and count PM_10_ and PM_0.1_ in outdoor settings using a predeposited vapor-condensed film, which could easily be applied to indoor settings. We have shown that a film deposited before particle aerosolization can form nanolenses around particles after they land on the film. Similar to work done where the film is deposited on top of the desired particles, a meniscus forms along the surface of the particle and enables detection of particles down to 100 nm by increasing the scattering cross section of the particle. Increasing the scattering cross section increases the signal of the particle in the lensfree reconstruction to a level above the noise. The strength of the signal enables sizing of the particles using a calibration code generated for the film thickness.

This device is competitive in terms of cost as well as performance, since few consumer devices are able to size and discriminate between PM_10_, PM_2.5_, and PM_0.1_ in a single device, making it well positioned to be adopted by citizens for personal air quality monitoring. Further improvements to the device would include the ability to send data over Bluetooth and automatic post-processing so that users could leave the device out in the desired monitoring environments and receive daily reports on the air quality or emergency reports if the detected air quality was hazardous.

## Notes

Drs. McLeod and Baker are inventors on intellectual property related to lensfree imaging.
